# Cultivation of earthworms and analysis of associated bacterial communities during earthworms’ growth using two types of agricultural wastes

**DOI:** 10.1186/s40643-024-00781-5

**Published:** 2024-07-09

**Authors:** Feng Qian, Fuzhi Lu, Liping Yang, Tingkao Li

**Affiliations:** 1 Guangxi Key Laboratory of Sericulture Ecology and Applied Intelligent Technology, School of Chemistry and Bioengineering, Hechi University, Hechi, 546300 China; 2 Guangxi Collaborative Innovation Center of Modern Sericulture and Silk, School of Chemistry and Bioengineering, Hechi University, Hechi, 546300 China; 3 Guangxi Colleges Universities Key Laboratory of Exploitation and Utilization of Microbial and Botanical Resources, School of Chemistry and Bioengineering, Hechi University, Hechi, 546300 China

**Keywords:** Silkworm excrement, Cow manure, Earthworm cultivation, Bacterial community

## Abstract

Earthworm cultivation can effectively promote the resource utilization of agricultural waste. The efficient utilization of agricultural waste by earthworms mainly depends on the microbial communities in the guts. This study used silkworm excrement and cow manure as substrates for earthworm cultivation and investigated the associated bacterial communities during earthworms’ growth. The survival rate of earthworms remained above 89% after 21 days of feeding with the two substrates. *Proteobacteria*, *Actinobacteria*, and *Firmicutes* constituted the predominant bacterial communities in earthworm growth, accounting for over 81% of the relative abundance in both guts and vermicompost. The bacteria richness and diversity in the foregut and midgut of earthworm were lower than those in the hindgut. The prediction function of intestinal bacterial communities of earthworms cultured with two substrates mainly involved biosynthesis, decomposition and energy production.

**Supplementary Information:**

The online version contains supplementary material available at 10.1186/s40643-024-00781-5.

## Highlights

Silkworm excrement and cow manure were used as substrates for earthworm cultivation.

Silkworm excrement, like cow manure, is a good substrate for earthworm cultivation.

*Proteobacteria*, *Actinomycetes* were the dominant bacteria during earthworms’ growth.

Earthworm activity and gut transit influence the abundance of dominant bacteria.

**Supplementary Information:**

The online version contains supplementary material available at 10.1186/s40643-024-00781-5.

## Introduction

Earthworms are important members of soil ecosystems that feed on humus and have a considerable capacity for processing waste without producing other pollutants. Consequently, they play critical roles in the decomposition of organic matter in terrestrial ecosystems, converting it into stable humus-like substances (i.e., vermicompost) that contain plant-available nutrients and abundant microorganisms that are beneficial for ecosystem health (Gusain & Suthar 2020). Vermicompost involves the biodegradation of organic matter via interactions between earthworms and microorganisms, with worm gut microbiomes significantly contributing to these processes (Suthar 2011). Specifically, gut-associated microorganisms provide several essential exogenous enzymes that aid in the rapid digestion of ingested organic components, while the foreguts and midguts digest and assimilate organic matter, with the hindguts then excreting it (Suthar 2010a, b).

Increasing attention has been paid in recent years to the important roles that earthworms exhibit in agricultural production cycles. Vermicompost or cultivation using agricultural waste and livestock manure has been investigated (Table 1), along with the associated microbial communities. Suthar used cattle manure to cultivate the earthworm *Allolobophora parva* (*Eisen*), which exhibited an individual biomass of up to 190.9 ± 0.07 mg, a maximum growth rate of 2.66, and a maximum weekly egg production of 26 ± 1.12 (Suthar 2011). In addition, Koubova et al. investigated the impact of earthworms (*Eisenia* spp.) on microorganisms in soil, compost, and cultivation, where they analyzed the microbial communities present within earthworm guts, fresh feces, and substrates (Koubova et al. 2015). The culturable microbial biomass in the gut and feces of earthworms was higher than that in the substrate. Wang et al. investigated changes in bacterial taxonomic characteristics and the potential functions of gut bacteria in earthworms (*Eisenia foetida*) that feed on cow manure (Wang et al. 2021). Feeding by earthworms reduced the abundances of Proteobacteria and Bacteroidetes but increased the abundances of *Actinobacteria*, *Chloroflexi*, and *Acidobacteria*. Earthworm guts are rich in microorganisms capable of metabolizing carbon and nitrogen compounds. Rupani et al. assessed the composting of organic waste from the campus of Islamic Azad University in Tehran, Iran, employing earthworms. Their study demonstrated that a waste system comprising 50% vegetable waste, 25% cow manure, and 25% waste paper yielded significant composting benefits and effectively mitigated *Salmonella* abundance (Rupani et al. 2023). Zhong et al. constructed a microbial community-earthworm system to evaluate the composting of feces and carbon-rich waste (rice straw (RS) and sawdust (S), and used it to investigate the effects of earthworms and carbon-rich waste on composting performance, and important microbial species (Zhong et al. 2023). The addition of RS or S promoted the growth and reproduction of earthworms, reduced the concentrations of volatile solids in substrates and *Escherichia coli* in feces, and significantly impacted the microbial community within the system phylogenetically. In addition, Devi et al. investigated the vermicomposting of lignocellulosic materials using two types of earthworms (*Eisenia foetida* and *Eudrilus eugeniae*) introduced at different feeding densities to evaluate the effects of different earthworm feeding densities on microbial community structures (Devi et al. 2023). Low-density *Eudrilus eugeniae* and high-density *Eisenia foetida* significantly increased soil microbial diversity and effectively regulated the metal restoration efficacy of vermicomposting systems. Srivastava et al. also conducted vermicomposting using poultry waste, rice straw, and cattle manure as substrates, specifically evaluating the effects of different substrate combinations, including cattle manure, cattle manure + rice straw, cattle manure + poultry waste, and cattle manure + poultry waste + rice straw. The combination of cattle manure + poultry waste + rice straw (1:1:1) subsequently exhibited the best vermicomposting effects (Srivastava et al. 2023).

**Table 1:** References on the cultivation of earthworms by various substrates

Earthworm species	Feeding substrate	Reference
*Perionyx excavatus*	soybean straw, wheat straw, maize stover, chickpea straw, city garbage	Manna et al. 1997
*Eudrillus euginae*	soil, cow dung and water hyacinth (1:1), partially dried neem leaves with kitchen waste (1:1)	Chakrabarty et al. 2009
*Allolobophora parva*	cattle dung	Suthar 2011
*Eisenia andrei*	cow, horse and pig manure	Aira et al. 2016
*Lumbricus terrestris*	barley and wheat straw, farmyard manure, cereal straw	Sizmur et al. 2017
*Eisenia foetida*	cow manure	Wang et al. 2021
*Eisenia foetida*	cattle manure, aquatic plant residues	Cui, et al., 2023
*Eisenia foetida*,* Eudrilus eugeniae*	lignocellulosic feedstock	Devi et al. 2023
*Eisenia foetida*	food waste, cow dung	Zheng et al. 2023
*Eisenia Foetida*	vegetable waste, cow manure, waste paper	Rupani et al. 2023
*Eudrilus eugeniae*,* Eisenia foetida*	poultry waste, rice straw, cattle manure	Srivastava et al. 2023

Silkworm excrement is a mixture of silkworm excrement, cocoon skin, and residual mulberry leaf debris, in addition to bran, straw, lime, and other materials added during silkworm cultivation (Wang et al. 2016). China is a major center of the silkworm industry, with abundant silkworm excrement resources and an annual output of up to 2.146 million tons. However, abundant silkworm excrement has been discarded carelessly, causing ecological environmental problems such as odor and water pollution. In addition, people have traditionally only used silkworm excrement as ordinary fertilizer directly on crops, thereby reducing its production value and benefits. However, increasing awareness of environmental protection and the development of silkworm excrement treatment technology on the harmless and resourceful development and use of this excrement. For example, silkworm excrement has been used as raw materials to extract chlorophyll, leaf protein, pectin, biochar, and other products, in addition to its use in livestock and poultry feed, organic fertilizer, biogas production, the cultivation of edible fungi, and other areas of agriculture. The main component of silkworm excrement is silkworm feces that can also be used for vermiculture. Wu explored the use of silkworm excrement for earthworm cultivation and achieved good cultivation results (Wu 2010). While studies have explored the composition and characteristics of silkworm excrement, its development and use have remained limited.

It is noteworthy that several studies have investigated the use of cow manure to raise earthworms and related microbial communities, but few studies have evaluated the use of silkworm excrement for raising earthworms. Furthermore, no studies have investigated intestinal microbial communities in silkworm excrement-raised earthworms, although these communities have important influences on their growth, reproduction, and decomposition of organic matter. Can silkworm excrement serve as a substrate for earthworms, similar to other animal waste such as cow manure? Are there discernible differences in the functional microbiota during the cultivation process? In contrast, this study aimed to explore earthworm cultivation using silkworm excrement and cow manure substrates. The impacts of the two substrates on earthworm cultivation and the related bacterial communities were investigated by detecting earthworm growth indicators, the bacterial communities in the substrates before cultivation, vermicompost after cultivation, and the fore-, mid-, and hindgut of earthworms after 21 days of cultivation.

## Materials and methods

### Substrates and earthworms

The silkworm excrement used in this study was obtained from the Silkworm Cultivation Farm in Hechi City, Guangxi Province, China, and the cow manure was collected from cattle farmers in Hechi City, Guangxi Province, China. Both silkworm excrement and cow manure underwent composting at room temperature (25–32 °C) for a period of 30 days. Throughout this process, the moisture content was maintained at approximately 60%, with the material being turned every 10 days to ensure adequate decomposition.

The earthworms (*Eisenia foetida*) were purchased from the Earthworm Factory in Jurong, Jiangsu Province, China. They were first acclimated for 3 days at room temperature in their original soil with a moisture of 60–70% before starting the experiment.

### Earthworm cultivation and sampling

**Fig. 1:**
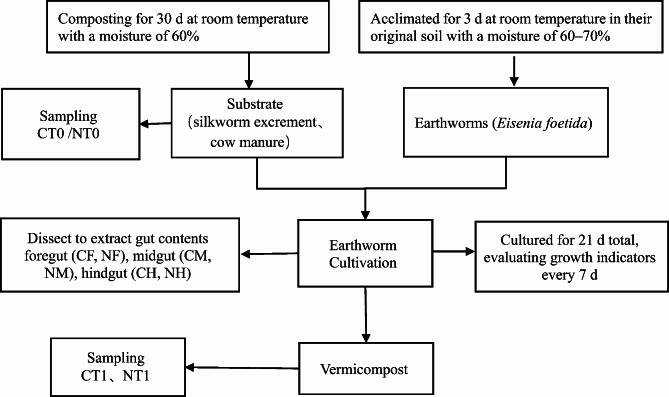
The flow chart of experimental design. CT0: silkworm excrement substrate; NT0: cow manure substrate; CT1: vermicompost (feeding on silkworm excrement); NT1: vermicompost (feeding on cow manure); CF, CM and CH: foregut, midgut, and hindgut contents of earthworm feeding on silkworm excrement respectively; NF, NM and NH: foregut, midgut, and hindgut contents of earthworm feeding on cow manure respectively

Six plastic boxes (300 × 200 × 150 cm) were prepared with multiple small holes (5 mm in diameter) around the box. Three of the plastic boxes were filled with 3 kg of silkworm excrement substrate and the other three were filled with 3 kg of cow manure substrate. The main nutrient contents of the silkworm excrement substrate (CT0) and the cow manure substrate (NT0) are shown in Table 2. After acclimation for 3 days, 300 healthy and vigorous earthworms weighing 0.30 g and measuring approximately 4.59 cm were selected for formal experiments. The 300 earthworms were randomly divided into the silkworm excrement group and the cow manure group, with 3 parallel samples included for each group, totaling 6 samples, with 50 earthworms in each sample. The earthworms were randomly added to the plastic boxes and then raised at room temperature with a moisture of 65%. After every 7 days of cultivation (over 21 days total), the earthworms in the plastic boxes were carefully removed to evaluate their growth indicators. The experimental design process was shown in Fig. 1. Silkworm excrement and cow manure substrate samples before raising earthworms (CT0 and NT0), silkworm excrement-earthworm manure samples after raising earthworms (CT1), and cow manure-earthworm manure samples after raising earthworms (NT1) were collected. After 21 days of cultivation, 100 earthworms were randomly selected from each group, and their intestinal tracts were aseptically dissected to extract their gut contents. These contents were divided into those from the foregut, midgut, and hindgut. The intestinal samples from the silkworm excrement group earthworms were identified as CF, CM, and CH respectively, while those from the cow manure group earthworms were identified as NF, NM, and NH respectively. The intestinal contents were sampled as previously described by Wang et al. (Wang et al. 2021).

**Table 2:** The nutrient content of silkworm excrement substrate and cow manure substrate

Sample	organic matter (%)	available nitrogen(mg/kg)	available phosphorus(mg/kg)	Available potassium(mg/kg)
CT0	55.2±2.3	62.8±1.7	84.1±2.9	210.3±4.3
NT0	39.5±2.9	58.3±0.8	79.5±1.7	196.4±3.1

CT0: silkworm excrement substrate; NT0: cow manure substrate

### Earthworm growth indicators

During sampling, the substrate in each plastic box was slowly poured into a tray to carefully select and count the surviving earthworms and cocoons. Earthworm surfaces were then washed with distilled water, followed by their placement in a clean petri dish for 5 min to allow the expulsion of undigested food. They were then washed again with distilled water and dried with filter paper to absorb surface moisture. Earthworm weights were measured using an electronic balance accurate to 0.001 g, with the worms immediately returned to boxes after weighing. The survival rate and daily weight multiple of the earthworms were calculated using the equations indicated below (Wang & Wang 2013).

1 $$\text {\:Earthworm\:survival\:rate}\:\left(\%\right)\hspace{0.17em}=\hspace{0.17em}N1/N0*100\%$$

2 $$\text{\:Daily\:weight\:multiple\:}=\:(G1-G0)/\left(G0*T\right)$$

where.

N_0_ = initial number of earthworms.

N_1_ = number of surviving initial earthworms after a certain period of cultivation.

G_0_ = initial weights of earthworms.

G_1_ = total weights of earthworms after a certain period of cultivation.

T = cultivation time.

In these formulas, the cultivation time was calculated in days (d) and earthworm weights were calculated in grams (g).

### Associated bacteria 16 S rRNA sequencing

DNA extraction, PCR amplification, Illumina MiSeq sequencing, and amplicon sequence processing were performed on all samples collected during earthworm cultivation. Microbial DNA was extracted from samples using a Fast DNA SPIN sequencing extraction kit (MP Biomedicals, USA) according to the manufacturer’s instructions. Partial 16S rRNA genes were then amplified, comprising the hypervariable V3-V4 regions, using the primers 338F (5’- ACTCCTACGGGAGGCAGCAG-3’) and 806R (5’-GGACTACHVGGGTWTCTAAT-3’). The PCR products were sequenced on an Illumina TruSeq platform according to standard protocols at Shanghai Personal Biotechnology Co., Ltd. (Shanghai, China).

### Taxonomy classification and statistical analysis

The Quantitative Insights into Microbial Ecology (QIIME 2) pipeline was employed to process the sequencing data. The sequences were subjected to pre-processing (quality-adjustment, barcode splitting), identification of amplicon sequence variants (ASV), taxonomic assignment, community comparisons, and statistical analyses. Taxonomic annotation was performed using the Greengenes database (Release 13.8, http://greengenes.secondgenome.com/) and the QIIME2 classify-sklearn algorithm (https://github.com/QIIME2/q2-feature-classifier). For each ASV feature sequence, taxonomic annotation was conducted using a pre-trained Naive Bayes classifier with default parameters in QIIME2. Alpha diversity analysis was performed using several indices, including Chao1, Shannon, Simpson. Using the non-rarefied ASV table, the “qiime diversity alpha-rarefaction” command was executed with the parameters “--p-steps 10 --p-min-depth 10 --p-iterations 10”, indicating a minimum rarefaction depth of 10. The “--p-max-depth” parameter was set to 95% of the lowest sequencing depth among all samples. The “qiime diversity alpha-rarefaction” command generated an alpha-rarefaction.qzv file, which was visualized at https://view.qiime2.org/ to plot rarefaction curves. Linear discriminant analysis (LDA) Effect Size (LEfSe) analyses were used to analyze differentially abundant taxa across groups using the default parameters. MetaCyc database analyses, phylogenetic investigation of the bacterial communities was evaluated using the reconstruction of unobserved states (PICRUSt2) to predict the metabolic pathways involved in the intestinal microbes (Li et al. 2021).

## Results and discussion

### Earthworm growth indicators

Silkworm excrement and cow manure substrates were used for cultivating earthworms, with surviving numbers, weights and reproduction measured on the 7th, 14th, and 21st days of cultivation (Table 3). After 21 days of cultivation, the survival rate of earthworms bred with silkworm excrement was 92.31%, while that of earthworms bred with cow manure was 88.46%. Given the high levels of survival in both substrates, both were suitable for cultivating earthworms. Comparison of the daily weight multiple and reproductive capacity of earthworms in the two substrates revealed that the use of cow manure as a substrate was slightly better than that of silkworm excrement, although these differences were not significant. In the investigation of the suitability of soybean straw, wheat straw, corn straw, chickpea straw, and municipal waste as food sources for *Perionyx excavatus*, variations in nutrient content among these organic wastes were identified as influencing their respective culture effects (Manna et al. 1997). In this study, though differences were observed in the nutritional contents of silkworm excrement and cow manure, with silkworm excrement having higher concentration of organic matter than cow manure, but the ratios of nitrogen, phosphorus, and potassium were basically similar in the two (Table 2). These similarities likely explain the lack of significant difference in their effectiveness for earthworm cultivation.

**Table 3:** The growth indicators of earthworm

Sample	Survival rate (%)	Daily weight multiple	No. of adults	No. of hatchlings	No. of cocoons
ES0	100	-	50	0	0
EC0	100	-	50	0	0
ES1	95±1	0.0292±0.0002	47±1	0	13±1
EC1	98±2	0.0378±0.0001	49±2	0	11±1
ES2	95±1	0.0277±0.0001	47±1	0	32±3
EC2	91±1	0.0378±0.0002	45±1	0	34±3
ES3	92±2	0.0209±0.0003	46±2	28±5	24±3
EC3	89±1	0.0275±0.0002	44±1	29±5	26±4

ES: earthworms feeding on silkworm excrement; EC: earthworms feeding on cow manure; The numbers 0,1,2,3 represent 0,7,14,21 days of vermiculture

### Bacterial community compositions

**Fig. 2:**
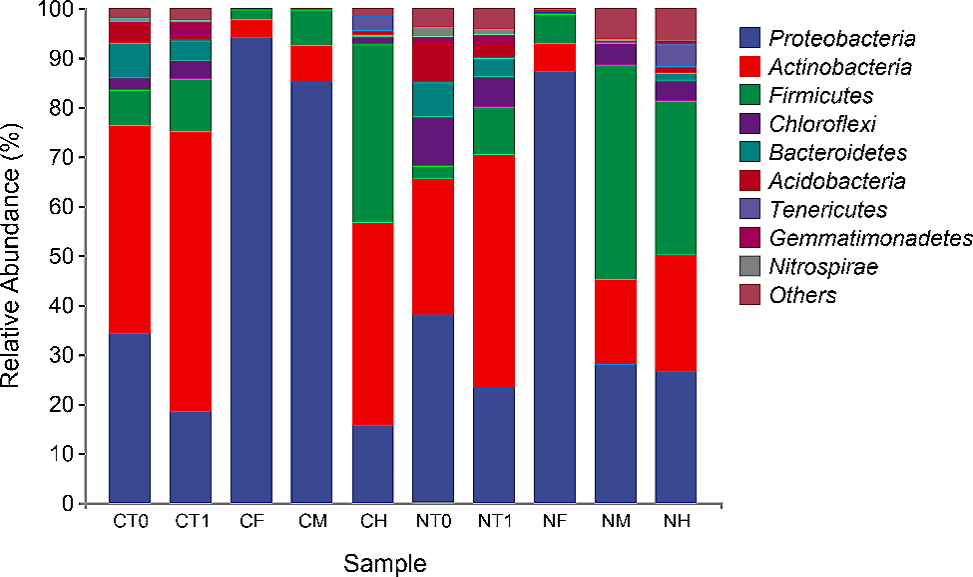
Relative abundances of the dominant bacterial communities at the phyla level. The percentage labels on the stacked bar chart represent the average relative abundances (top 9) of the phyla in each sample group, *n* = 3. CT0: silkworm excrement substrate; NT0: cow manure substrate; CT1: vermicompost (feeding on silkworm excrement); NT1: vermicompost (feeding on cow manure); CF, CM and CH: foregut, midgut, and hindgut contents of earthworm feeding on silkworm excrement respectively; NF, NM and NH: foregut, midgut, and hindgut contents of earthworm feeding on cow manure respectively

The bacterial communities associated with earthworm cultivation are shown in Fig. 2. The nine most abundant microbial phyla accounted for over 93% of the total ASV (amplicon sequence variants) abundances. The dominant bacterial communities in the silkworm excrement and cow manure substrates (CT0 and NT0) in addition to the silkworm excrement-earthworm manure and cow manure-earthworm manure (CT1 and NT1) were similar, primarily comprising *Proteobacteria*, *Actinomycetes*, and *Firmicutes*, albeit with different abundances. Comparison of the bacterial communities in the substrates and earthworm manure revealed that *Proteobacteria*, *Bacteroidetes*, and *Acidobacteria* exhibited significantly reduced abundances during transit through the earthworm gut, while *Actinobacteria* and *Firmicutes* exhibited increased abundances. The dominant microorganisms in the silkworm excrement substrate exhibited relatively similar abundances in the foreguts and midguts, although larger differences were observed in hindgut abundances. In contrast, the dominant microorganisms in the foreguts of earthworms fed with cow manure substrate exhibited larger differences in abundances compared to the midguts and hindguts. *Proteobacteria* dominated the CF, CM, and NF communities with abundances of 94%, 85%, and 87%, respectively. However, their abundances decreased to 16%, 28%, and 27% in the CH, NM, and NH communities, respectively. The relative abundances of *Actinobacteria* and *Firmicutes* significantly increased in both midguts and hindguts of earthworms fed with either substrate.

Previous studies have shown that earthworms guts act as a selective biofilter, selectively stimulating and inhibiting ingested microorganisms (Thakuria et al. 2010). Due to varying *in*-situ conditions of different earthworm gut segments and competition between gut microorganisms, the selective effects during transit through the earthworm gut may differ. The results of the present study indicated that the gut microorganisms of earthworms fed with two kinds of substrates exhibited significantly different abundances of dominant microorganism during transit through the gut. The abundances of *Proteobacteria* in the foreguts and midguts of the earthworms fed on silkworm excrement, and in the foreguts of the earthworms fed on cow manure was very high, but were significantly lower in the hindguts. *Proteobacteria* is the largest bacterial phylum and contains several important bacterial groups, some of which have excellent biodegrading capacities for the decomposition and use of various organic compounds. After ingesting silkworm excrement and cow manure from their environment, earthworms decompose organic matter with the help of intestinal microorganisms. The organic matter content in the foreguts of earthworms was higher compared with midguts and hindguts. Moreover, the unique anaerobic environment in the intestine likely stimulated the accumulation of *Proteobacteria*. *Actinobacteria* and *Firmicutes* were also the dominant microbial phyla in the earthworm gut, and their abundance increased in the middle and posterior intestines. Some *Actinobacteria* and *Firmicutes* have excellent biodegradability capacities and are important antibiotic-producing strains that can decompose various organic compounds and use them for secondary metabolism to produce antibiotics (Aira et al. 2016). Antibiotics produced by *Actinomycetes* and *Firmicutes* accumulate in the midguts and hindguts of earthworms, leading to decreased abundances of some antibiotic-sensitive strains that themselves gradually become dominant. Previous studies have shown that food resources could cause changes in gut bacterial communities of earthworms (Thakuria et al. 2010). Although the dominant bacterial communities in silkworm excrement and cow manure substrates were similar, *Chloroflexi* abundances differed. *Chloroflexi* in cow manure substrates accounted for about 10% of the total community, which is much higher than 2.5% of the silkworm excrement substrates. *Chloroflexi* enters bodies through earthworm feeding. Although *Chloroflexi* have low abundances in the foregut due to the intestinal environment and other dominant communities, their abundances increase in the hindgut and extend to vermicompost. *Chloroflexi* abundances in the cow manure feeding group were significantly higher than in the silkworm excrement feeding group, consistent with *Chloroflexi* abundances in the substrates. Research indicated that the bacterial communities in earthworm vermicompost were closely associated with the bacterial community composition present in the food consumed by earthworms during the cultivation (Aira et al. 2016). In this study, bacterial communities in the vermicompost from the two substrates used for cultivating earthworms were very similar to those in the respective cultivation substrates. However, since dominant bacterial communities in both silkworm excrement and cow manure substrates are fundamentally similar, significant differences were not observed between the bacterial communities in the vermicompost produced from silkworm excrement and that from cow manure.

### Microbial community diversity

The Chao1 and Observed species indices are measures of microbial community richness, with higher values indicating greater community richness, while the Shannon and Simpson indices are measures of microbial community diversity, with higher values indicating greater community diversity. The trends in the Chao1, Observed species, Shannon, and Simpson indices for samples from both substrates used for earthworm cultivation nearly universally followed the trends of: CT0 > CT1 > CH > CM > CF, NT0 > NT1 > NH > NM > NF (Table 4). With the exception of the Simpson index in the cow manure substrate-cultivated earthworm samples, which followed the trend of NT0 > NH > NM > NT1 > NF, the values for NT0, NT1, NM, and NH were all similar. These results indicate that earthworm activity in the cultivation substrates reduce the richness and diversity of bacteria therein, while the digestion of earthworms greatly affected bacterial richness and diversity, resulting in lower bacterial community richness and diversity in the foregut and midgut compared to the hindgut. These results are similar to previous studies of earthworms fed with cow manure, which observed that microbial alpha diversity decreases in the foregut and midgut during transit through the earthworm gut, followed by an increase in diversity in the hindgut (Wang et al. 2021). Additionally, research has discovered that foregut and midgut extracts from earthworms possess strong selective bactericidal activity, while hindgut extracts do not (Byzov et al. 2007; Khomyakov et al. 2007). Therefore, microorganisms that survive foregut and midgut stimulation begin to rapidly grow and reproduce in the hindgut, leading to significant increases in the richness and diversity of bacterial communities therein.

**Table 4:** Microbial community alpha diversity index data

Sample	Chao1	Observed species	Shannon	Simpson
CT0	7072.62	6400.0	10.0638	0.993738
CT1	4815.01	4371.1	8.58704	0.971469
CF	466.139	419.2	0.922187	0.150138
CM	915.239	780.6	1.72478	0.308849
CH	2038.75	1916.1	6.95807	0.937682
NT0	7288.35	7249.6	11.4092	0.99847
NT1	6770.7	6641.5	10.3831	0.991841
NF	1598.57	1387.4	2.27079	0.328988
NM	3378.33	3224.8	9.73623	0.995488
NH	4903.95	4538.6	10.1337	0.995969

CT0: silkworm excrement substrate; NT0: cow manure substrate; CT1: vermicompost(feeding on silkworm excrement); NT1: vermicompost (feeding on cow manure); CF, CM and CH: foregut, midgut, and hindgut contents of earthworm feeding on silkworm excrement respectively; NF, NM and NH: foregut, midgut, and hindgut contents of earthworm feeding on cow manure respectively

### Differential gut microbial community of earthworms

**Fig. 3:**
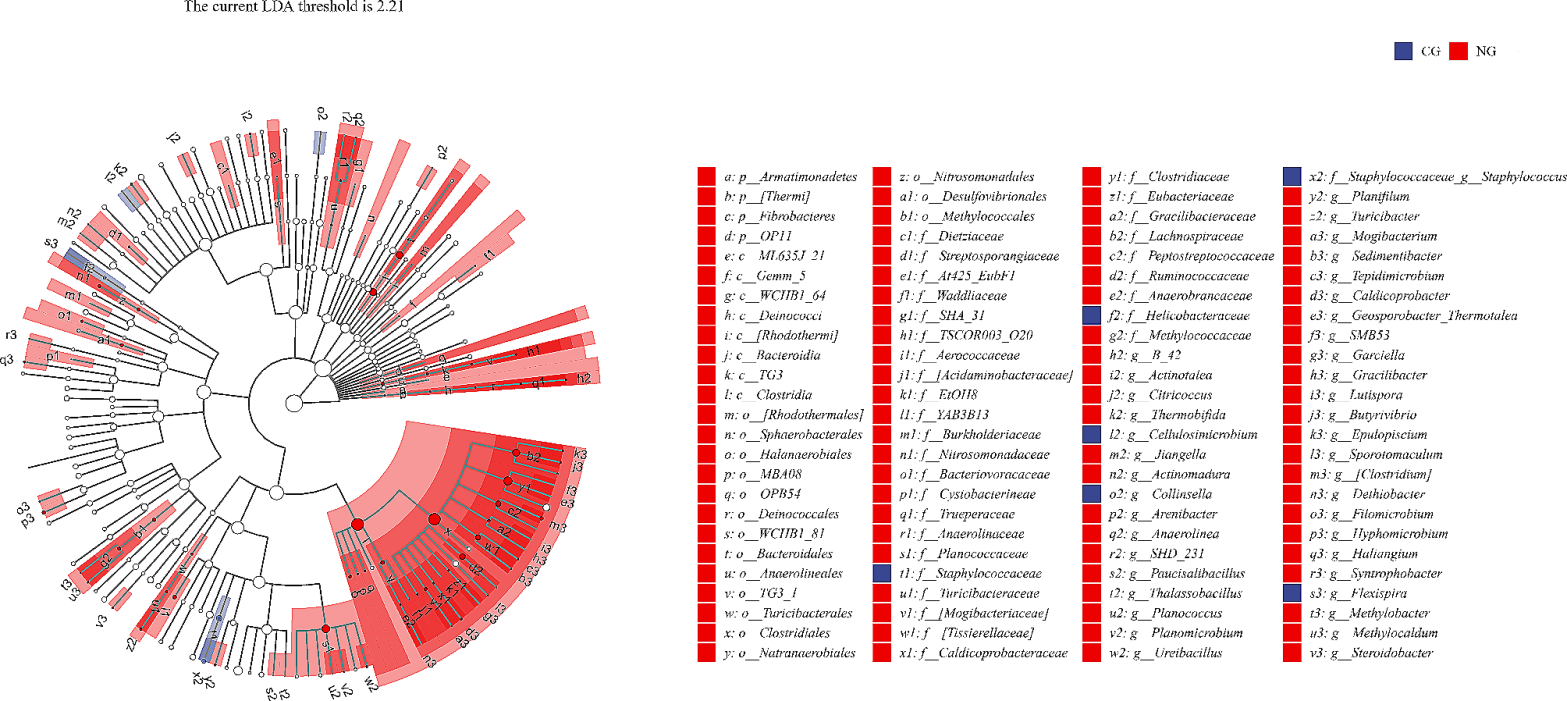
Differential taxon between the two groups of gut microflora based on classification hierarchy tree. The taxonomic branch diagram shows the taxonomic hierarchy of the main taxa from phylum to genus (from inner ring to outer ring) in the sample community. The node size corresponds to the average relative abundance of the taxon; the hollow nodes represent the taxon with no significant difference between groups, while the nodes with other colors indicate that these taxa show significant differences between groups, and the abundance of these taxa is higher in the grouped samples represented by this color (blue and red represent earthworm feeding on silkworm excrement group and earthworm feeding on cow manure group, respectively). Letters identify the names of taxa with significant differences between groups

To determine the bacterial communities with significant differences in abundance between two groups, Linear discriminant analysis (LDA) Effect Size (LEfSe) analyses were used to analyze the data (Fig. 3). A total 4 phyla, 8 classes, 16 orders, 31 families, and 41 genera were enriched in the cow manure cultivation group. The primary taxa enriched in this group were the Firmicutes and *Proteobacteria*, including the genera *Clostridium*, *Turicibacter*, *Haliangium*, and *Hyphomicrobium*. Only 2 families and 4 genera were observed in the gut microflora of earthworms from the silkworm excrement cultivation group, representing significantly higher numbers than the microbial abundances of the cow manure cultivation group, among which the main microbial groups were *Staphylococcacea*e including *Staphylococcus* and *Bacillus*.

According to the LEfSe analyses, the abundances of some bacterial communities were significantly different in the guts of earthworms fed with different substrates, but there were not many bacterial communities with large differences, which is consistent with previous studies. Study assessing the gut microbial community of earthworms have found that the influence of food on the gut microbial community of earthworms was less significant compared to the effects of the ecosystem, habitat, and other factors (Thakuria et al.2010). In this study, the cultivation conditions of earthworms in the two groups were consistent except for different substrates, so the differences in intestinal communities between the earthworms in the two groups were not very significant.

### Differences in gut microbial metabolism pathways

**Fig. 4:**
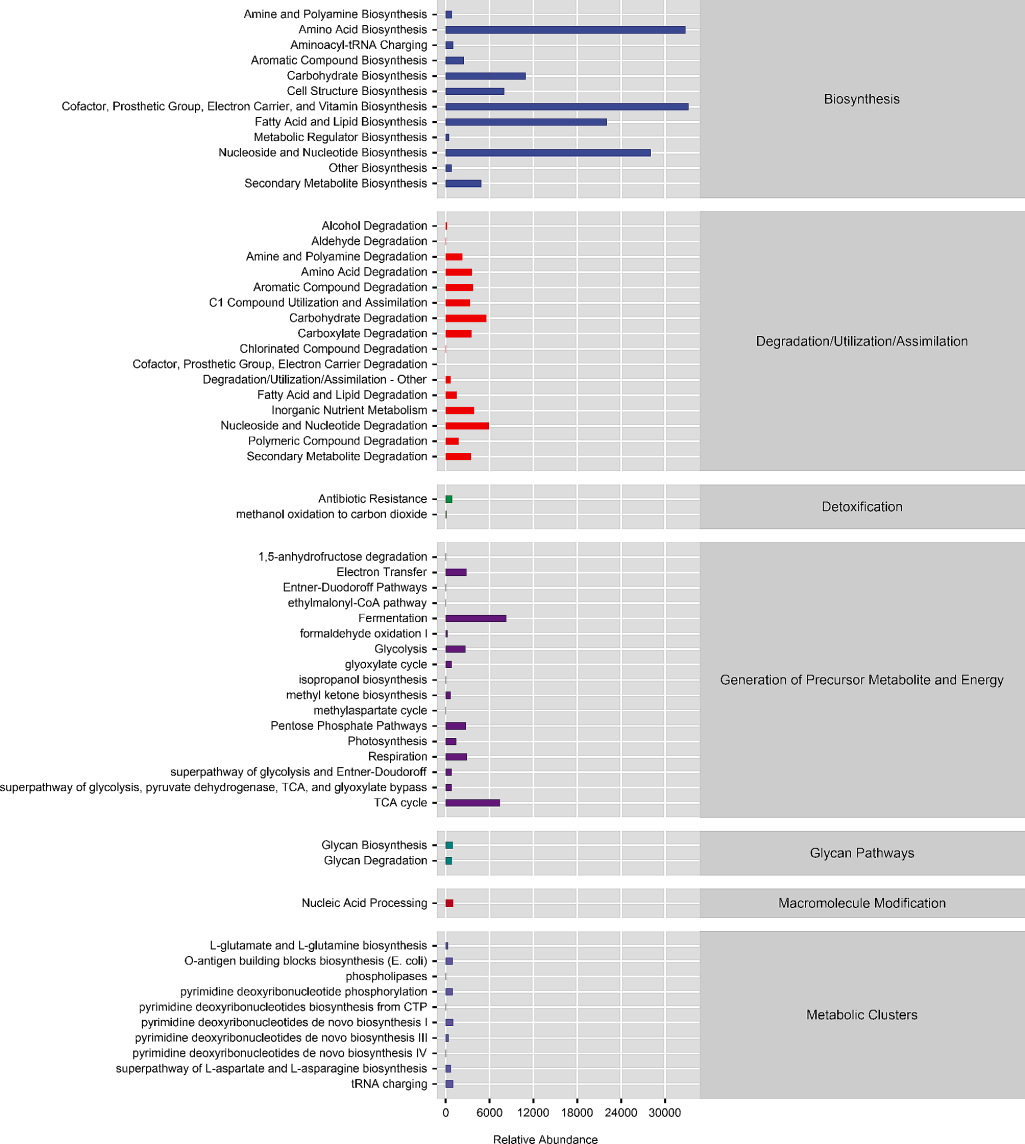
Predicted KEGG secondary functional pathway abundance map. The abscissa represents the abundance of functional pathways, the ordinate represents the functional pathways of the second classification level of KEGG (Kyoto Encyclopedia of Genes and Genomes), the rightmost box represents the first level to which the pathways belong

Based on 16 S rRNA gene sequence analyses and MetaCyc database analyses, phylogenetic investigation of the bacterial communities was evaluated using the reconstruction of unobserved states (PICRUSt2) to predict the metabolic pathways involved in the intestinal microbes of earthworms. Predictions indicated that the intestinal microorganisms primarily participated in 60 pathways, including biosynthesis, degradation/utilization/assimilation, detoxification, generation of precursor metabolite and energy, glycan pathways, macromolecule modification, and metabolic clusters (Fig. 4). These pathways were primarily associated with the metabolic categories of amino acid biosynthesis, carbohydrate biosynthesis and degradation, fatty acid and lipid biosynthesis, nucleoside and nucleotide biosynthesis, aromatic compound degradation, and fermentation of the host.

From these analyses, differences in predicted MetaCyc metabolic pathways between the silkworm excrement cultivation communities and the cow manure cultivation communities were analyzed. With the cow manure cultivation group as the control, several functions were enriched in the silkworm excrement cultivation group, including peptidoglycan biosynthesis II (staphylococci). In addition, methanogenesis from acetate, biotin biosynthesis II, coenzyme B biosynthesis (a total of 3 pathways) were significantly unenriched. The peptidoglycan biosynthesis II (staphylococci) pathway was significantly enriched in the silkworm excrement cultivation group samples, indicating a higher level of peptidoglycan synthesis in the metabolites of microorganisms in that group. The peptidoglycan biosynthesis II(Staphylococcus) pathway is a crucial biosynthesis pathway for Gram-positive bacteria that not only provides bacteria with essential nutrients, but also protects them from external environments (Moran et al. 2009). The methanogenesis from acetate pathway occurs under anaerobic conditions and is the final step in the decomposition of organic matter by microorganisms (Pan et al. 2016). The intestinal tract of earthworms provides an anaerobic environment for microorganisms that enables microorganisms to initiate this metabolic pathway and further convert acetic acid generated by the decomposition of organic matter in the intestine into methane and carbon dioxide, thereby completing the degradation of organic matter. The biotin biosynthesis II pathway results in biotin (vitamin H) synthesis in microorganisms and plants. Biotin is a coenzyme involved in carboxylation reactions that are essential for synthesizing of fatty acids and the degrading amino acids. Coenzyme B is used in the electron transport chain in some bacteria and helps conserve energy, thereby enabling adaptation to environmental stresses and becoming essential for microbial survival and reproduction. When cultivating earthworms with two kinds of substrates, differences in the functional metabolic pathways of intestinal communities will affect the decomposition of organic matter in the intestine and the absorption of nutrients, thereby affecting host growth and reproduction. The results of this study indicate that the intestinal metabolic pathways of earthworms cultivated with different substrates are affected by growth conditions; however, this difference is not significant.

## Conclusions

The survival rate, daily weight multiple and reproductive capacity of earthworms cultivated with silkworm excrement were similar to those of earthworms cultivated with cow manure, indicating that silkworm excrement can be used as a good substrate for earthworm cultivation. During earthworm cultivation, the bacterial community structures in the substrates, earthworm guts, and vermicompost were found to be similar, only the abundances of dominant bacterial communities varied, suggesting interchangeability among bacterial communities during earthworm cultivation. Earthworm activities and gut transit can also influence changes in dominant microbial populations. Notable differences were observed in the abundances of certain metabolic pathways of the functional bacterial communities of earthworm guts cultivated with the two substrates, indicating that food sources can also impact metabolic pathways of the gut bacterial communities, and thus affect the degradation and utilization of the substrate by earthworms. The results provide a reference for the cultivation of earthworms by silkworm excrement and the utilization of agricultural waste by earthworms.

### Electronic supplementary material

Below is the link to the electronic supplementary material.

Supplementary Material 1

## Data Availability

Data will be made available on request.
