# Supplementary figures and images for: Cultivation of earthworms and analysis of associated bacterial communities during earthworms’ growth using two types of agricultural wastes
Source: Bioresour Bioprocess. 2024 Jul 9;11(1):66. doi: 10.1186/s40643-024-00781-5 (PMC11233487; doi:10.1186/s40643-024-00781-5)

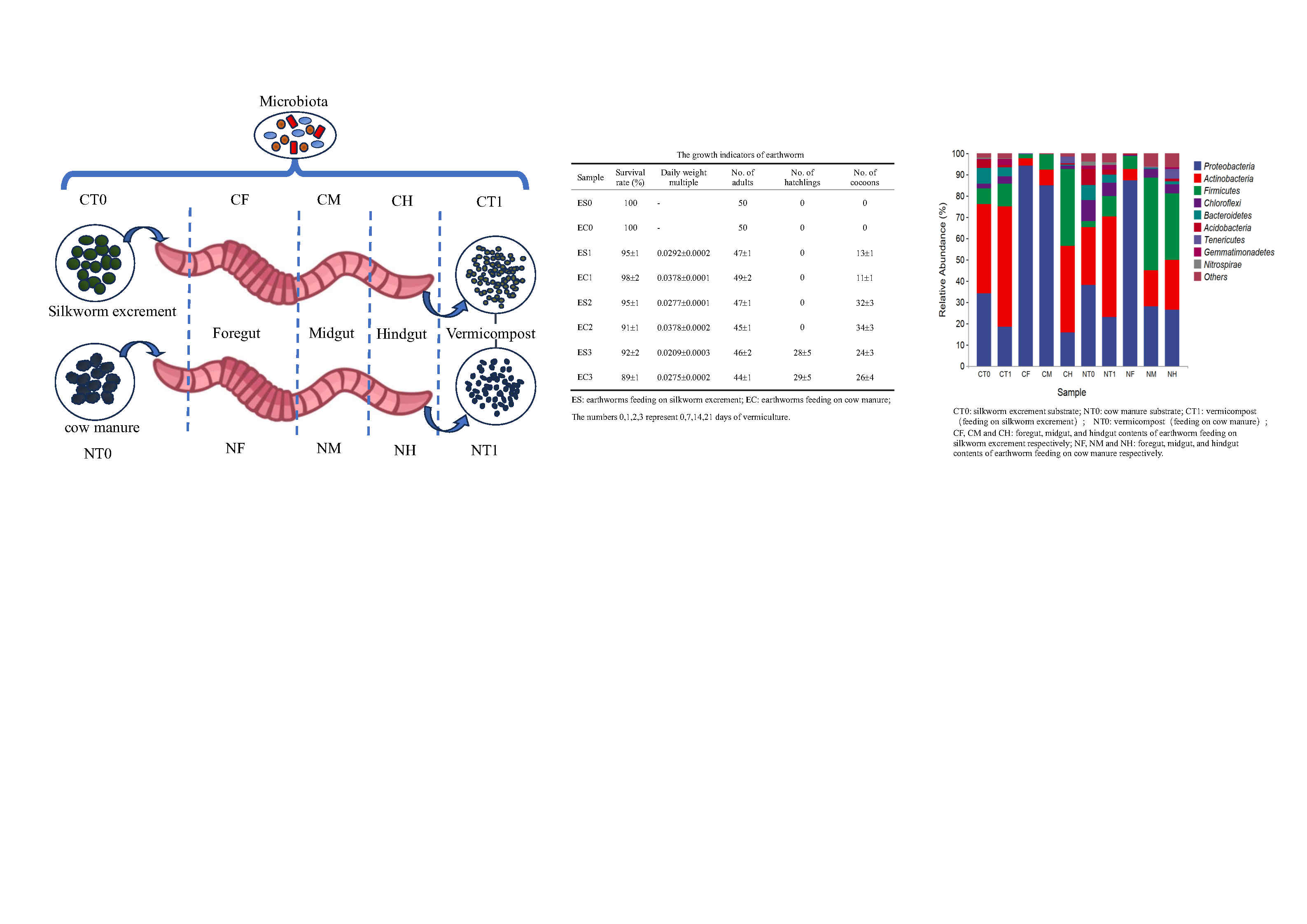

Supplement: Supplementary file 1 — Supplementary Material 1 [file 40643_2024_781_MOESM1_ESM.jpg]
